# Supplementary material for: Workflow sharing with automated metadata validation and test execution to improve the reusability of published workflows
Source: Gigascience. 2023 Feb 22;12:giad006. doi: 10.1093/gigascience/giad006 (PMC9944229; doi:10.1093/gigascience/giad006)
Supplement: giad006_Response_to_Reviewer_Comments_Original_Submission [file giad006_response_to_reviewer_comments_original_submission.pdf]

Author's Response To Reviewer Comments

Close

Point-by-point response to reviewers' comments

GIGA-D-22-00187
Workflow sharing with automated metadata validation and test execution to improve the reusability of published workflows
Hirotaka Suetake; Tsukasa Fukusato; Takeo Igarashi; Tazro Ohta
GigaScience

We would like to thank reviewers for their thoughtful comments and constructive feedbacks. We have added a summary figure and some additional paragraphs to make things clear, and made changes in the figures from the previous version of the manuscript.

Our responses and explanations to the issues pointed by each reviewer are follows.

Reviewer #1

> 1. Based on your survey of existing systems, could you possibly make a figure or table that showcases the features supported/not supported by these different systems, including yours?

Response: Thank you for the suggestion that can highlight the contribution of our work. We added the Table 2 in the Result section to compare the characteristics of WorkflowHub, Dockstore, nf-core, and a yevis-based registry. We compared them with three aspects; diversity, reliability, and usability of registered workflow.

> 2. Thoughts on security/cost safeguards? Perhaps beyond the scope, but it does seem like a governing group needs to define some limits to the testing resources and be able to enforce them. If I am a bad actor and programmatically open up 1000 PRs of expensive jobs, I'm not sure what would happen. Actions and artifact storage aren't necessarily free after some limit.

Response: We first had security concerns with the functionality in GitHub. Then, to solve problems of offensive actions by using malicious users via Pull Requests, we use GitHub's first-time contributor restriction. In this system, users (who first contribute to the repository) cannot perform a GitHub action without permission from the administrator. That is, the owners can easily reject "Pull Requests" from suspicious users, i.e., avoiding possible security issues.

As for the resource limitation of GitHub Actions, we solve the problem by using an external WES. In addition to a lack of resources, GitHub Actions may not be sufficient for testing workflows that only work on specific system configurations, such as GPUs or job schedulers. In such cases, the registry owner can check the testing portability by running the test on a remote WES instance. We added sentences to the Discussion section about the limitations of GitHub resources, the incident possibilities, and solutions to prevent them.

We added the following sentences to the Discussion section:

Automatic testing with GitHub actions may also cause the issue of computational resource shortage. To extend the capability of testing, Yevis has the option to specify the location of an external WES endpoint to run the test, which also enables the testing with a specific computational request such as GPUs or job schedulers.

> 3. What is the flow for simply updating to a new version of an existing workflow? (perhaps this could be in your docs, not necessarily this manuscript).

Response: We appreciate the comment about the version updates, which is one of the critical features of our implementation. The system identifies a workflow by its remote URL of the primary workflow in the metadata. Therefore, if the user modifies the metadata without changing the URL and bumps up the version information, the system recognizes that they are different versions of the same workflow. Users can use different versions of the workflow by specifying the TRS endpoint with a path including version information, such as "tools/0d2ae4c2-fe4c-48f7-811a-ac277776533e/versions/1.0.0".

Users can upgrade the registered workflow by performing the following steps.

1. Edit the existing yevis-metadata.yml file.
2. Bump the workflow version in the metadata.
3. Run tests using yevis-cli and send a PR to the repository.
4. Follow the same steps as adding a new workflow, and the new version will be published.

We added the above instruction to the online documentation.

> 4. CWL is an example of a workflow language that developers can extend to create custom "hints" or "requirements". For example, seven bridges does this in cavatica where a user can define aws spot instance configs etc. WDL has properties to config GCP images. It seems like in these cases, tests should only be defined to work when running "locally" (not with some scheduler/specific cloud env). But the author's do mention that tests will first run locally on the user's environment, so that does kind of get around this.

Response: As we see the importance of the testing portability, we implemented the system with a strict specification. Developers can push the workflows that passed the local test to the registry, and the owners can merge those that passed the remote test. However, as the reviewer points out, specific compute-dependent tests exist in actual use cases and cannot be ignored. Allowing tests to be run outside the developer's own environment can solve the testing portability problem, i.e. the testing on a computing environment that anyone can access, even if not on GitHub. Therefore, the ability of our GitHub action to request tests to external WES instances can solve the problem. However, another problem is preparing a WES instance that can correctly run the tests for that workflow. We believe it is the responsibility of developers who want to share their workflows to prepare an environment where anyone can execute the tests correctly. Perhaps the preparation of a testing environment is something that the community should discuss.

> 5. For the "findable" part of FAIR, how possible is it to have "tags" of sort associated with a wf record so things can be more findable? I imagine when there is a large repository of many workflows, being able to easily narrow down to the specific domain interest you have could be helpful.

Response: It is crucial to curate the metadata in the registry with tags or ontology terms that increase findability. The findability issue is a downside of the de-centralized registry model. Although it is possible to add tags as part of the TRS response specification, tagging with uncontrolled vocabulary by each workflow developer will not improve the findability. We believe that a centralized search index is in need to distinguish workflows from other similar or similar but different workflows available online. Therefore, a possible strategy is to register the metadata in a centralized registry such as WorkflowHub, while the workflow body is hosted and test runs on GitHub/Zenodo, as we proposed.

We have added this point as a weakness of the de-centralized model in the Discussion as following:

Another challenge for the proposed distributed registry model is the findability of workflows. In the model where each developer is responsible for their content, the use of appropriate terms for describing workflow metadata can be an issue. A possible solution to improve the findability of workflows in distributed registries is to collect metadata in a centralized registry to curate them and create the search index. However, this will require a further challenge to distinguish the collected workflows using only metadata.

Reviewer #2

Main concern

> I have one major gripe though, blocking acceptance: The choice to only support GitHub for hosting. There is a growing problem in the research world that more and more research is being dependent on the single commercial actor GitHub, for seemingly no other reason than convenience. Although GitHub to date can be said to have been a somewhat trustworthy player, there is no guarantee for the future, and ultimately this leaves a lot of research in an unhealthy dependenc on this single platform. As a small note of a recent change, is the proposed removal of the promise to not track its users (see https://github.com/github/site-policy/pull/582).
> A such a central infrastructure component for research as a workflow registry has an enormous responsibility here, as it may greatly influence the choices of researchers in the future to come, because of encouragement of what is "easier" or more convenient to do with the tools and infrastructure available.
> With this in mind, I find it unacceptable for a workflow registry supporting open science and open source work to only support one commercial provider.
> The authors mention that technically they are able to support any vendor, and also on-premise setups, which sounds excellent. I ask the authors to kindly implement this functionality. Especially the ability to run on-premises registries is key to encourage research to stay free and independent from commercial concerns.

Response: We understand the reviewer's concern about the lock-in risk to a commercial service well. It is not our desire to be locked into a specific service, and we are aware of the risk that these services (and every other service run by a vendor) may become unavailable to everyone. However, at the same time, it is impractical for us to implement and provide the "perfect lock-free" version of the system with production-level quality as well. We would like to explain the reasons below.

In this study, we aimed to achieve two major objectives: the independency from computational resources maintained by individual developers, and the automation to reduce the developers' tasks that make workflows reusable.

For the first objective, the academic communities know from their experience how difficult it is to maintain one's server for decades, at least for most of the developers in research. We think the main reason why many researchers rely on GitHub is simple, they do not need to maintain their git servers. This is also why we chose to use services run by vendors while realizing the risk of lock-in.

For the second objective, we had to retrieve the metadata of workflows automatically. Therefore, we used the GitHub API as a core component, which is not straightforward to replace with a plain on-premise git server.

Therefore, we have to give up these two strong benefits if we wanted to provide the same functionalities with a possible on-premise Yevis implementation. We still can think of the lock-free version of the registry, however, it will look like a completely different one as follows:

1. Workflow developers manually create metadata.yml and run the tests by hand using WES.
2. Developers submit the metadata to the registry administrator using a communication channel such as a mailing list.
3. The administrator runs tests using WES based on the received metadata.yml and reviews them.
4. Upload the reviewed workflow to any file server and publish it.

With these steps, partially supported by the current yevis-cli, one can have a workflow registry completely vendor lock-free. However, it is not as far efficient as the system using GitHub and Zenodo.

We believe our contribution is not the system itself but rather the distributed registry model in which communities can have their platforms by automating the quality assurance tasks. We hope that GitHub will not become evil, but if it does, we will re-implement our system with other services.

To clarify the benefit and the risk of vendor lock-in, and to explain the form of the system without those services, we added the sentences to the discussion section as follows:

Yet we currently provide only the implementation that depends on those two services while it is technically possible to build a Yevis registry in an on-premise server. It is because the vendor-free system can only achieve part of our objectives to provide the effortless management of a registry. Here, we recognize a trade-off of building an academic tool using services run by commercial vendors, which requires further discussion in the communities.

Minor concerns

> 1. I think the manuscript is a missing citation to this key workflow review, as a recen overview of the bioinformatics workflows field, for example together with the current citation [6] in the manuscript:
> Wratten, L., Wilm, A., & Göke, J. (2021). Reproducible, scalable, and shareable analysis pipelines with bioinformatics workflow managers. Nature methods, 18(10), 1161-1168.
> https://www.nature.com/articles/s41592-021-01254-9

Response: Thank you for pointing out the important citation. We added the citation as suggested.

> 2. Although it might not have been the intention of the authors, the following sentence sounds unneccessarily subjective and appraising, without data to back this up (rather this would be something for the users to evaluate):
> The Yevis system is a great solution for research communities that aim to share their workflows and wish to establish their own registry as described.
> I would rather expect wording similar to:
> "The Yevis system provides a [well-needed] solution for ..."
> ... which I think might have been closer to what the authors intended as well.

Response: We thank the precise suggestion to the statement. We edited the sentence as suggested.

Reviewer #3

> One of my main concerns is that this works presents a technical implementation but lacks an architecture diagram or a big picture that would clarify the contributions of this work with respect to the features provided by the external platforms (GitHub, Zenodo). This would also help the reader understand how the solution proposed can be reused with possibly other services.

Response: We agree with the reviewer's concern that the system's overall architecture is not clear to the readers. Although we expected that the Figure 1 in the original submission shows the overall procedure with the components we used, it focuses on the steps of the submission, not the environment where the system is running. We added a new diagram as Figure 1 to indicate the components where each process is running and how they are connected each other.

> It is also hard to understand the technical solutions when target users and their typical needs have not been clearly stated beforehand. It seems to be very complicated for non-developers to use or operate Yevis, especially when testing workflows through the "GitHub actions" infrastructure. Is the Yevis platform only targeting workflow developers ? It was not easy to understands the benefits offered to research communities aimed at sharing/reusing workflows.

Response: Firstly, we would like to thank the reviewer for pointing out that it is not clear about the system's target user is. We agree that it was implicit in the manuscript that the technical level of the users expected to use the system. We expect workflow developers as the target users of the Yevis system, i.e., developers and researchers with the skills to design and implement arbitrary workflows using the workflow language. However, the procedure for publishing workflows using the Yevis system does not require any of the difficulties noted by the reviewer. The repository template used in the Yevis setup procedure already has the GitHub action for workflow testing that the system automatically executes when a pull request is

received. Thus, the users do not need to write any GitHub actions but prepare the test data for their workflows and write their metadata. As indicated in the new system overview figure, many procedures exist to publish. However, the system automates them except for the writing of metadata and the administrator's review. We believe that this labor-saving automation is the contribution of this research.

To clarify the target users, we added a sentence to the Background section as follows:

The system expects developers and researchers who design workflows using workflow languages as users, although it does not require advanced computer skills to operate the system.

> Regarding the background section, GA4GH-TRS is not introduced while mentioned as part of the results. It's hard for the reader to understand how the use of this standard contributes to better workflow sharing (metadata ?) or better reuse (tests?).

Response: We added the sentence to the second paragraph of the System design section as follows:

The TRS defines the specification of computational tool/workflow metadata representation, including workflow's URI, used language, version, etc. It ensures the interoperabilities among different tool/workflow registries and enables workflow engines to retrieve the information to execute a workflow maintained at a remote server, which improves reusability of published workflows.

> Other workflow registries such as WorkflowHub or NF-Core have been described in the background section but a dedicated state-of-the-art section would have allowed the authors to provide more details on the positioning of Yevis. Some related works should also be part of the analysis such as BIAFLOWS also providing a benchmarking environment, or OpenEBench.

Response: We added the Table 2 to compare the existing registries, including the WorkflowHub, nf-core, and Dockstore, to compare their characteristics with a Yevis-based registry. We reviewed the BIAFLOWS and OpenEBench and we recognized their features of tool/workflow collections and evaluations. However, their objectives are to provide a summary to compare different computational tools for better tool selection, which is a slightly different scope from the aim of this study to publish workflow with testing results for better trust. Therefore, we exclude them from the table, but we thank the reviewer for pointing out the relevance of our system with those excellent community resources.

> The live deployment URL of the Yevis system should be provided in the paper so that readers can browse/reuse already registered workflow. The link provided in the source code repository only shows 4 workflows and not the DAT2 workflow used in the "proof of concept" section. Is the system limited to some workflow engines ? Would it be possible to register and test Galaxy workflows for instance ?

Response: In the original manuscript, we mentioned the registry URL of the DAT2-cwl registry in the reference section (37), but we recognized it was not clear to the readers. We added the URL to the DAT2-cwl registry (https://github.com/pitagora-network/yevis-DAT2-cwl) to the "sharing workflows using Yevis" section and the repository of yevis-cli. The registry with four workflows is the DDBJ workflow registry, which is different from the one we have shown as a demo in the manuscript. We also have corrected the resources mentioned in the manuscript.

The yevis system itself does not have a limitation on workflow engines but requires a WES endpoint to test the given workflow. Thus, if we had a WES instance that consumes a Galaxy workflow the system can accept a Galaxy workflow. Currently, we have a WES instance available for Common Workflow Language, Nextflow, Workflow Description Language, and Snakemake.

> Regarding tests of workflows, the files associated to HiSAT2 on the Pitagora workflow were not accessible (404 not found). I also had some difficulties when trying to inspect the results of the automated tests in the GitHub actions. For this workflow, [Add workflow: Pitagora CWL - Download SRA · ddbj/workflow-registry@89961a4 · GitHub](https://github.com/ddbj/workflow-registry/actions/runs/2256980244), the logs were expired and thus no more accessible. This highlights the challenge of relying on external computing services to run possibly long and costly executions, even with

test data.

Response: We thank the reviewer for pointing out a ctirical issue of the current implementation. We missed this end-of-life of the GitHub actions artifact during our development phase. This GitHub action artifact is for two objectives, and we expect this limitation does not affect the current Yevis procedure. First, the artifact is supposed for the review by the registry administrator. If 90 days have passed since the automatic execution and the test log is missing, the admin can re-run the action and review the artifact again. Second, the artifact is also valuable as a workflow run provenance. For this purpose, we have added a function in the WES instance to generate ResearchObject-crate (RO-crate), a standard specification to record the research artifact, to let the Yevis system store the test log in the Zenodo repository.

For long and costly workflow execution problem, the system has an option to run tests with the external WES endpoint shown in the new summary figure. It brings the availability issue of the testing instance but can solve the problem of the resource limitation of the GitHub actions.

> Regarding the validation of metadata, very few informations are provided. Are all metadata fields mandatory ? are some fields recommended ? Which kind of validation is performed ? How the result of validation is returned to users ? Regarding the metadata themselves, how do they comply with community emerging standards such as Bioschemas or RO-crate ? At the time of the review, it was not possible to find any semantic annotations in the Yevis web page, thus limiting the discoverability and the interoperability of workflows descriptions.

Response: For each field in the metadata, the system performs a type check, license check, URL validation, remote file existence, etc. We added the information on these validation items to the CLI's GitHub repository. (https://github.com/sapporo-wes/yevis-cli#validate) The CLI checks these metadata items written by the user, and if it finds an invalid field, the CLI outputs an error message and prompts the user to correct it. As the reviewer points out, our specification for workflow metadata overlaps with some of the community-developed standards. Currently, for workflow test run logs, we are working with the community to apply the workflow run crate under development by the RO-crate community. However, as we prioritize the readability and editability of Yevis metadata, the approach could be to adopt a proprietary format and then convert it to a standard by the community.

Because of technical limitations in the current implementation, embedding tags in the pages is not practical to improve findability. As we implemented the Yevis-web in the JavaScript Single Page Application framework, it is not possible to embed different annotation tags in the HTML source of each workflow page.

However, we believe workflow findability should not be left to individual workflow developers but should be a centralized effort by a central registry. Therefore, we would like to enhance the findability of workflows by having individual communities manage their workflows with Yevis while a centralized registry such as WorkflowHub collects these metadata.

> Finally, the discussion and future works sections could be enriched to address for instance.
> - the scalability of the approach with possibly long or costly tasks when testing workflows
> - the interoperability of this platform with other registries
> - the genericity of the approach (is it applicable in the context of other scientific disciplines)
> - the use of this platform to compare or benchmark workflow executions based on predefined test datasets

Response: We would like to thank the reviewer's suggestion to make the Discussion section more constructive.
We added the following topics in the Discussion section to reflect the reviewers' feedback.

- Using external WES endpoint to overcome the resource limitation of GitHub actions: also brings the concern of availability (3rd paragraph)
- The automation of quality control procedure using CI/CD of the public services: its limitation and possibility (3rd paragraph)
- The interoperability of the Yevis registry and possibilities of coexistence with the other centralized

registries for findability (6th paragraph)

We excluded the topic of benchmarking because we think it can confuse the readers as if the Yevis system can use for that purpose. We expect benchmarking feature should be provided on a different layer than that of the registry.

Minor comments

> Figures 2 and 7 seems to be very similar. Only one should be kept.

Response: We agree with the reviewer's assessment. To avoid the confusion, we removed the Figure 7 which showed the metadata file modified by the yevis system. We remained Figure 2 and added the explanation that the metadata will be modified.

> How are test specified, is the specification generic enough? How does it support multiple workflow engines?

Response: Yevis's specification of the test run conforms to the GA4GH WES specification. In yevis-cli, the option --wes-location specifies the WES instance on which the system runs the test. As WES implementations usually have a single workflow engine, users need to use a WES capable of executing the workflow's language. As an exception, Sapporo, one of the WES implementations we have developed, provides users the option to specify a workflow engine from several engines covering CWL, WDL, Nextflow, and Snakemake. The yevis-cli command uses Sapporo via Docker if users have no WES preference.

We added sentences to emphasize the option to specify an external WES for testing instance as follows:

Automatic testing with GitHub Actions may also cause the issue of computational resource shortage. To extend the capability of testing, Yevis has the option to specify the location of an external WES endpoint to run the test, which also enables the testing with a specific computational request such as GPUs or job schedulers.

> The paragraph on decentralization in the discussion is confusing. All workflow executions seem to be centralized on the GitHub infrastructure with no control on data or computation placement. The same happens for data on the Zenodo infrastructure.

Response: Thank you for your comments. We think there is a misunderstanding about the term decentralization. In the submission stage, we described "Yevis can promote the concept of a distributed workflow registry model that underlies the specifications of the GA4GH Cloud Work Stream," so this paragraph claim is the idea of decentralization of workflow registries, not computation placement. Since we emphasize that the communities have an option to build their own registry, not relying on a centralized registry such as WorkflowHub or nf-core, we added a sentence to clarify the idea as follows:

In the distributed workflow registry model, researchers have the option to build their own workflow registry, rather than submitting to a centralized registry.

As mentioned above, the computation also can be decentralized using the external WES instances.

> DAT2-cwl is not registered in the live deployment of Yevis

Response: The original manuscript has referred external GitHub repositories and the resources, which may confuse readers. We added the URL in the sentence along with the citation to lead the readers to the appropriate resource.

Close
