# Supplementary material for: Workflow sharing with automated metadata validation and test execution to improve the reusability of published workflows
Source: Gigascience. 2023 Feb 22;12:giad006. doi: 10.1093/gigascience/giad006 (PMC9944229; doi:10.1093/gigascience/giad006)
Supplement: giad006_Response_to_Reviewer_Comments_Revision_1 [file giad006_response_to_reviewer_comments_revision_1.pdf]

Author's Response To Reviewer Comments

Close

GIGA-D-22-00187
Workflow sharing with automated metadata validation and test execution to improve the reusability of published workflows
Hirotaka Suetake; Tsukasa Fukusato; Takeo Igarashi; Tazro Ohta
GigaScience

We would like to thank the reviewers again for their constructive feedback. The revised manuscript highlighted the changes in red color. Our responses to the issues and concerns pointed out by each reviewer are as follows.

> Reviewer #2:
> In line with my previous comment, I find it unacceptable for such a core component for the research enterprise at large as a workflow registry, to promote reliance on a single, closed source vendor (GitHub), without an open and free fallback solution, especially for a publication that focuses so heavily on, and has gained a reputation for, promoting open science practices.

Again, we agree with the reviewer's concern about the strong dependencies on GitHub, a company's platform. To provide our proposed methods without such dependencies, we prepared the scripts and protocol for building a Yevis repository with no dependencies on any third-party web services. While the alternate version lacks some original features such as the review interface or external resource validation, the procedures are simple as follows:

1. Write workflow metadata [Submitter]
2. Run workflow test locally [Submitter]
3. Submit metadata to the registry admin [Submitter]
4. Run workflow test on the registry side [Admin]
5. Generate TRS response and edit [Admin]
6. Publish the generated TRS response [Admin]
7. (Optional) Deploy Yevis-web on an on-premise server [Admin]

The on-premise version documentation and the source code are hosted on our web server (https://data.dbcls.jp/~inutano/yevis/yevis_on_premise.zip). We added the description about the on-premise version in the third paragraph of Discussion.

> Reviewer #3:
> The proposed platform targets both workflow sharing and testing. It is explicitly stated in the abstract: "the validation and test are based on the requirements we defined for a workflow being reusable with confidence". It is clear in the paper that tests are realized through the GitHub CI infrastructure, possibly delegated to a WES workflow execution engine. Although I inspected Figure 3 as well as the wf_params.json and wf_params.yml provided in the demo website. It doesn't seem to be enough to answer questions such as: how are specified tests ? How can a user inspect what has been done during the testing process ? What is evaluated by the system to assess that a test is successful ?
> I tried to understand what was done during the testing process but the test logs are not available anymore ([Add workflow: human-reseq: fastqSE2bam · ddbj/workflow-registry@19b7516 · GitHub](https://github.com/ddbj/workflow-registry/actions/runs/2257134260))

We recognize the complexity when one wants to follow the testing details after a workflow was registered. The answers to the reviewer's questions are as follows:

> how are specified tests ?

The current workflow testing performed by the Yevis system is a simple test run with example input data. As shown in Fig 3, metadata.yml has a "testing" field that contains a list of input files for the registering workflow. Yevis runs the workflow by using these files as its input, then checks the final execution status.

We added the sentences to explain the testing method as follows:

As a workflow testing, Yevis runs a workflow with specified input data files and check the final execution status. If the run is completed successfully, Yevis considers the workflow passed the test.

> How can a user inspect what has been done during the testing process ?

As the reviewer indicated, the log file of the GitHub action will be expired after 90 days of its execution. To keep the log file, the repository owner needs to register to a GitHub Pro account (payment required). Initially, we expected that only the registry admin checks the log file for the purpose of submission screening. In that case, the admin can run the action again to generate the log file after the expiration date. However, as the reviewer did, we recognize the needs that the workflow users may want to check the workflow testing log files. For this use case, we have a future plan to implement a feature to store the RO-crate, a community standard for scientific provenance, as a provenance of workflow testing. By generating an RO-crate from the test run and storing it on GitHub, users can inspect the testing.

We added the sentence to show the future plan as below:

The registry maintainer can check the testing log as an artifact file on GitHub action. However, the file will expire 90 days after execution. To keep the provenance of the test log, we aim to improve the system to have a function to record the test procedure in a standard format, such as RO-crate.

> What is evaluated by the system to assess that a test is successful ?

As explained above, the current testing is a simple workflow run with the specified input data. An automatic evaluation of a workflow run is challenging. We defined another problem here, and we are working on a solution called Tonkaz.

We edited the sentence in the Discussion to show the ongoing project to evaluate the testing result as below:

One of the challenges is how workflow developers write the workflow testing. Currently, Yevis tests the workflows by running them with the specified input files and evaluates the execution status. However, the execution status only shows the successful completion of the computing process, which does not ensure the workflow produced the outputs as expected. Therefore, the test can pass even if the input files are not the ones that reflect the real use cases. The evaluation of the outputs is not as simple as checking the output file identities, because some workflows can produce outputs with subtle differences which do not change the biological interpretation. For example, the correct outputs of the same workflow may not be identical because of the tools using heuristic algorithms or regularly updated databases. We are challenging this problem in a separate project and aim to incorporate the results into our system in the future
https://www.biorxiv.org/content/10.1101/2022.10.11.511695v2

> Regarding the findability of the workflows, in line with FAIR principles, the discussion mentions a possible solution which would consists in hosting and curating metadata in another database. To tackle workflow discoverability between multiple systems, accessible on the web, we could expect that the Yevis registry exposes semantic annotations, leveraging Schema.org (or any other controlled vocabulary) for instance. This would also make sense since EDAM ontology classes are referred to in the Yevis metadata file (https://ddbj.github.io/workflow-registry-browser/#/workflows/65bc3bd4-81d1-4f2a-8886-1fbe19011d81/versions/1.0.0).

Although we understand the reviewer's intent, it is not feasible in the current implementation to add semantic annotations to improve the discoverability of a Yevis-based registry. The main body of Yevis registries is the raw JSON files that are served as TRS responses via GitHub pages. These files are data and require an external index for internet search. The Yevis web browsing interface, on the other hand, is a lightweight JavaScript (JS) application. Since the application has no data of its own and dynamically consumes JSON files from the TRS API, the page cannot embed the annotation in advance. This is a tradeoff in the implementation method: we chose to build the app as a JS application because it can be easily hosted on GitHub pages, which reduces deployment costs. Therefore, we think the best way to improve the discoverability of the workflows hosted on a Yevis Workflow is to keep the main workflow files on GitHub, but submit the metadata to a central registry like the WorkflowHub. We are working on this idea with the WorkflowHub folks so that it can be implemented in the future.

Close
