# Supplementary material for: Workflow sharing with automated metadata validation and test execution to improve the reusability of published workflows
Source: Gigascience. 2023 Feb 22;12:giad006. doi: 10.1093/gigascience/giad006 (PMC9944229; doi:10.1093/gigascience/giad006)
Supplement: giad006_Response_to_Reviewer_Comments_Revision_2 [file giad006_response_to_reviewer_comments_revision_2.pdf]

Author's Response To Reviewer Comments

Close

We have updated the manuscript following the editorial direction as follows:
- Registered software on SciCrunch.org and received RRID. IDs are shown in the availability section of the manuscript
- Removed text highlights
- Added authors' ORCIDs

Close
