# Supplementary material for: Workflow sharing with automated metadata validation and test execution to improve the reusability of published workflows
Source: Gigascience. 2023 Feb 22;12:giad006. doi: 10.1093/gigascience/giad006 (PMC9944229; doi:10.1093/gigascience/giad006)
Supplement: giad006_Reviewer_1_Report_Original_Submission — Kyle Hernandez, Ph.D. -- 8/2/2022 Reviewed [file giad006_reviewer_1_report_original_submission.pdf]

Reviewer Report

Title: Workflow sharing with automated metadata validation and test execution to improve the reusability of published workflows

Version: Original Submission **Date:** 8/2/2022

Reviewer name: Kyle Hernandez, Ph.D.

Reviewer Comments to Author:

Suetake et. al designed and developed a system to publish, validate, and test public workflows utilizing existing standards and integration with modern CI/CD tools. Their design wasn't myopic, they relied heavily on their own experiences, work from GA4GH, and interacting with the large workflow development communities. They were inspired by the important work from Goble et. al that applies the FAIR standards to workflows. As someone who had a long history of workflow engine development, workflow development, and workflow reusability/sharing experience I greatly appreciate this work. There are still unsolved problems, like guidelines on how to approach writing tests for workflows for example, but their system is one level above this and focuses on ways to automate the validation, testing, reviewing/governance, and publishing into a repository to greatly reduce unexpected errors from users. I looked through the source code of their rust-based client, which was extremely readable and developed with industry-level standards. I followed the read me to setup my own repositories, configure the keys, and deploy the services successfully on the first walk through. That speaks to the level of skill, testing, and effort in developing this system and is great news for users interested in using this. At some level it can seem like a "proof of concept", but it is one that is also usable in production with some caveats. The concept is important and implementing this will hopefully inspire more folks to care about this side of workflow "provenance" and reproducibility. There are so many tools out there for CI/CD that is often poorly utilized by academia and I appreciate the author's showing how powerful they can be in this space. The current manuscript is fine and will be of great interest to a wide ranging set of readers, I only have some non-binding suggestions/thoughts that could improve the paper for readers:

1. Based on your survey of existing systems, could you possibly make a figure or table that showcases the features supported/not supported by these different systems, including yours?
2. Thoughts on security/cost safeguards? Perhaps beyond the scope, but it does seem like a governing group needs to define some limits to the testing resources and be able to enforce them. If I am a bad actor and programmatically open up 1000 PRs of expensive jobs, I'm not sure what would happen. Actions and artifact storage aren't necessarily free after some limit.
3. What is the flow for simply updating to a new version of an existing workflow? (perhaps this could be in your docs, not necessarily this manuscript).
4. CWL is an example of a workflow language that developers can extend to create custom "hints" or "requirements". For example, seven bridges does this in cavatica where a user can define aws spot instance configs etc. WDL has properties to config GCP images. It seems like in these cases, tests should only be defined to work when running "locally" (not with some scheduler/specific cloud env). But the author's do mention that tests will first run locally on the user's environment, so that does kind of get

around this.
5. For the "findable" part of FAIR, how possible is it to have "tags" of sort associated with a wf record so things can be more findable? I imagine when there is a large repository of many workflows, being able to easily narrow down to the specific domain interest you have could be helpful.

Methods

Are the methods appropriate to the aims of the study, are they well described, and are necessary controls included? Choose an item.

Conclusions

Are the conclusions adequately supported by the data shown? Choose an item.

Reporting Standards

Does the manuscript adhere to the journal's guidelines on minimum standards of reporting? Choose an item.

Choose an item.

Statistics

Are you able to assess all statistics in the manuscript, including the appropriateness of statistical tests used? Choose an item.

Quality of Written English

Please indicate the quality of language in the manuscript: Choose an item.

Declaration of Competing Interests

Please complete a declaration of competing interests, considering the following questions:

- Have you in the past five years received reimbursements, fees, funding, or salary from an organisation that may in any way gain or lose financially from the publication of this manuscript, either now or in the future?
- Do you hold any stocks or shares in an organisation that may in any way gain or lose financially from the publication of this manuscript, either now or in the future?
- Do you hold or are you currently applying for any patents relating to the content of the manuscript?
- Have you received reimbursements, fees, funding, or salary from an organization that holds or has applied for patents relating to the content of the manuscript?
- Do you have any other financial competing interests?

- Do you have any non-financial competing interests in relation to this paper?

If you can answer no to all of the above, write 'I declare that I have no competing interests' below. If your reply is yes to any, please give details below.

I declare that I have no competing interests

I agree to the open peer review policy of the journal. I understand that my name will be included on my report to the authors and, if the manuscript is accepted for publication, my named report including any attachments I upload will be posted on the website along with the authors' responses. I agree for my report to be made available under an Open Access Creative Commons CC-BY license (http://creativecommons.org/licenses/by/4.0/). I understand that any comments which I do not wish to be included in my named report can be included as confidential comments to the editors, which will not be published.

Choose an item.

To further support our reviewers, we have joined with Publons, where you can gain additional credit to further highlight your hard work (see: https://publons.com/journal/530/gigascience). On publication of this paper, your review will be automatically added to Publons, you can then choose whether or not to claim your Publons credit. I understand this statement.

Yes Choose an item.
