# Supplementary material for: Workflow sharing with automated metadata validation and test execution to improve the reusability of published workflows
Source: Gigascience. 2023 Feb 22;12:giad006. doi: 10.1093/gigascience/giad006 (PMC9944229; doi:10.1093/gigascience/giad006)
Supplement: giad006_Reviewer_2_Report_Original_Submission — Samuel Lampa, PhD -- 8/13/2022 Reviewed [file giad006_reviewer_2_report_original_submission.pdf]

Reviewer Report

Title: Workflow sharing with automated metadata validation and test execution to improve the reusability of published workflows

Version: Original Submission **Date:** 8/13/2022

Reviewer name: Samuel Lampa, PhD

Reviewer Comments to Author:

The Yevis manuscript makes a good case for the need to be able to easily set up self-hosted workflow registries, and the work is a laudable effort.
From the manuscript, the implementation decisions seem to be done in a very thoughtful way, using standardized APIs and formats where applicable (Such as WES).
The manuscript itself is very well written, with a good structure, close to flawless language (see minor comment below) and clear descriptions and figures.
Main concern
I have one major gripe though, blocking acceptance: The choice to only support GitHub for hosting. There is a growing problem in the research world that more and more research is being dependent on the single commercial actor GitHub, for seemingly no other reason than convenience. Although GitHub to date can be said to have been a somewhat trustworthy player, there is no guarantee for the future, and ultimately this leaves a lot of research in an unhealthy dependenc on this single platform. As a small note of a recent change, is the proposed removal of the promise to not track its users (see https://github.com/github/site-policy/pull/582).
A such a central infrastructure component for research as a workflow registry has an enormous responsibility here, as it may greatly influence the choices of researchers in the future to come, because of encouragement of what is "easier" or more convenient to do with the tools and infrastructure available.
With this in mind, I find it unacceptable for a workflow registry supporting open science and open source work to only support one commercial provider.
The authors mention that technically they are able to support any vendor, and also on-premise setups, which sounds excellent. I ask the authors to kindly implement this functionality. Especially the ability to run on-premises registries is key to encourage research to stay free and independent from commercial concerns.
Minor concerns
1. I think the manuscript is a missing citation to this key workflow review, as a recen overview of the bioinformatics workflows field, for example together with the current citation [6] in the manuscript: Wratten, L., Wilm, A., & GÃ¶ke, J. (2021). Reproducible, scalable, and shareable analysis pipelines with bioinformatics workflow managers. Nature methods, 18(10), 1161-1168. https://www.nature.com/articles/s41592-021-01254-9
2. Although it might not have been the intention of the authors, the following sentence sounds unneccessarily subjective and appraising, without data to back this up (rather this would be something

for the users to evaluate):
> The Yevis system is a great solution for research communities that aim to share their workflows and wish to establish their own registry as described.
I would rather expect wording similar to:
"The Yevis system provides a [well-needed] solution for ..."
... which I think might have been closer to what the authors intended as well.
Wishing the authors best of luck with this promising work!

Methods

Are the methods appropriate to the aims of the study, are they well described, and are necessary controls included? Choose an item.

Conclusions

Are the conclusions adequately supported by the data shown? Choose an item.

Reporting Standards

Does the manuscript adhere to the journal's guidelines on [minimum standards of reporting?]() Choose an item.

Choose an item.

Statistics

Are you able to assess all statistics in the manuscript, including the appropriateness of statistical tests used? Choose an item.

Quality of Written English

Please indicate the quality of language in the manuscript: Choose an item.

Declaration of Competing Interests

Please complete a declaration of competing interests, considering the following questions:

- Have you in the past five years received reimbursements, fees, funding, or salary from an organisation that may in any way gain or lose financially from the publication of this manuscript, either now or in the future?
- Do you hold any stocks or shares in an organisation that may in any way gain or lose financially from the publication of this manuscript, either now or in the future?
- Do you hold or are you currently applying for any patents relating to the content of the manuscript?

- Have you received reimbursements, fees, funding, or salary from an organization that holds or has applied for patents relating to the content of the manuscript?
- Do you have any other financial competing interests?
- Do you have any non-financial competing interests in relation to this paper?

If you can answer no to all of the above, write 'I declare that I have no competing interests' below. If your reply is yes to any, please give details below.

I declare that I have no competing interests

I agree to the open peer review policy of the journal. I understand that my name will be included on my report to the authors and, if the manuscript is accepted for publication, my named report including any attachments I upload will be posted on the website along with the authors' responses. I agree for my report to be made available under an Open Access Creative Commons CC-BY license (http://creativecommons.org/licenses/by/4.0/). I understand that any comments which I do not wish to be included in my named report can be included as confidential comments to the editors, which will not be published.

Choose an item.

To further support our reviewers, we have joined with Publons, where you can gain additional credit to further highlight your hard work (see: https://publons.com/journal/530/gigascience). On publication of this paper, your review will be automatically added to Publons, you can then choose whether or not to claim your Publons credit. I understand this statement.

Yes Choose an item.
