# Supplementary material for: Workflow sharing with automated metadata validation and test execution to improve the reusability of published workflows
Source: Gigascience. 2023 Feb 22;12:giad006. doi: 10.1093/gigascience/giad006 (PMC9944229; doi:10.1093/gigascience/giad006)
Supplement: giad006_Reviewer_3_Report_Original_Submission — Alban Gaignard -- 8/31/2022 Reviewed [file giad006_reviewer_3_report_original_submission.pdf]

Reviewer Report

Title: Workflow sharing with automated metadata validation and test execution to improve the reusability of published workflows

Version: Original Submission **Date:** 8/31/2022

Reviewer name: Alban Gaignard

Reviewer Comments to Author:

This paper introduces the YEVIS workflow registry which aims at better sharing computational workflow by leveraging workflow metadata, testing datasets, as well as large-scale production infrastructures such as GitHub and Zenodo.

The main issue identified by the authors is the cost for workflow developers to publish and maintain reusable workflows. The contribution of the paper is the description of a system composed by i) a command-line software tool to assist users when registering workflows, and ii) a web application aimed at browsing registered workflows. These two components benefit from the GitHub infrastructure to automate workflow testing and from Zenodo to store and identify data with DOIs. The system is evaluated based on an example workflow.

General comments

Through the development of registries, the paper try to address very timely issues faced by large scientific communities. Researcher are more and more encouraged to publish their research artifacts online, but it is still difficult to make them discoverable and to concretely reuse them, especially for computational workflows.

Although the authors provide a landscape analysis of existing workflow languages, systems, and identify clearly the gaps between strongly and more lightly curated workflow registries, the quality of the paper should be improved.

One of my main concerns is that this works presents a technical implementation but lacks an architecture diagram or a big picture that would clarify the contributions of this work with respect to the features provided by the external platforms (GitHub, Zenodo). This would also help the reader understand how the solution proposed can be reused with possibly other services.

It is also hard to understand the technical solutions when target users and their typical needs have not been clearly stated beforehand. It seems to be very complicated for non-developers to use or operate Yevis, especially when testing workflows through the "GitHub actions" infrastructure. Is the Yevis platform only targeting workflow developers ? It was not easy to understands the benefits offered to research communities aimed at sharing/reusing workflows.

Regarding the background section, GA4GH-TRS is not introduced while mentioned as part of the results. It's hard for the reader to understand how the use of this standard contributes to better workflow sharing (metadata ?) or better reuse (tests ?) .

Other workflow registries such as WorkflowHub or NF-Core have been described in the background section but a dedicated state-of-the-art section would have allowed the authors to provide more details on the positioning of Yevis. Some related works should also be part of the analysis such as BIAFLOWS

also providing a benchmarking environment, or OpenEBench.
The live deployment URL of the Yevis system should be provided in the paper so that readers can browse/reuse already registered workflow. The link provided in the source code repository only shows 4 workflows and not the DAT2 workflow used in the "proof of concept" section. Is the system limited to some workflow engines ? Would it be possible to register and test Galaxy workflows for instance ?
Regarding tests of workflows, the files associated to HiSAT2 on the Pitagora workflow were not accessible (404 not found). I also had some difficulties when trying to inspect the results of the automated tests in the GitHub actions. For this workflow, [Add workflow: Pitagora CWL - Download SRA Â· ddbj/workflow-registry@89961a4 Â· GitHub](https://github.com/ddbj/workflow-registry/actions/runs/2256980244), the logs were expired and thus no more accessible. This highlights the challenge of relying on external computing services to run possibly long and costly executions, even with test data.
Regarding the validation of metadata, very few informations are provided. Are all metadata fields mandatory ? are some fields recommended ? Which kind of validation is performed ? How the result of validation is returned to users ? Regarding the metadata themselves, how do they comply with community emerging standards such as Bioschemas or RO-crate ? At the time of the review, it was not possible to find any semantic annotations in the Yevis web page, thus limiting the discoverability and the interoperability of workflows descriptions.
Finally, the discussion and future works sections could be enriched to address for instance
- the scalability of the approach with possibly long or costly tasks when testing workflows
- the interoperability of this platform with other registries
- the genericity of the approach (is it applicable in the context of other scientific disciplines)
- the use of this platform to compare or benchmark workflow executions based on predefined test datasets
Minor comments
- Figures 2 and 7 seems to be very similar. Only one should be kept.
- How are test specified, is the specification generic enough ? How does it support multiple workflow engines ?
- The paragraph on decentralization in the discussion is confusing. All workflow executions seem to be centralized on the GitHub infrastructure with no control on data or computation placement. The same happens for data on the Zenodo infrastructure.
- DAT2-cwl is not registered in the live deployment of Yevis

Methods

Are the methods appropriate to the aims of the study, are they well described, and are necessary controls included? Choose an item.

Conclusions

Are the conclusions adequately supported by the data shown? Choose an item.

Reporting Standards

Does the manuscript adhere to the journal's guidelines on minimum standards of reporting? Choose an item.

Choose an item.

Statistics

Are you able to assess all statistics in the manuscript, including the appropriateness of statistical tests used? Choose an item.

Quality of Written English

Please indicate the quality of language in the manuscript: Choose an item.

Declaration of Competing Interests

Please complete a declaration of competing interests, considering the following questions:

- Have you in the past five years received reimbursements, fees, funding, or salary from an organisation that may in any way gain or lose financially from the publication of this manuscript, either now or in the future?
- Do you hold any stocks or shares in an organisation that may in any way gain or lose financially from the publication of this manuscript, either now or in the future?
- Do you hold or are you currently applying for any patents relating to the content of the manuscript?
- Have you received reimbursements, fees, funding, or salary from an organization that holds or has applied for patents relating to the content of the manuscript?
- Do you have any other financial competing interests?
- Do you have any non-financial competing interests in relation to this paper?

If you can answer no to all of the above, write 'I declare that I have no competing interests' below. If your reply is yes to any, please give details below.

I declare that I have no competing interests

I agree to the open peer review policy of the journal. I understand that my name will be included on my report to the authors and, if the manuscript is accepted for publication, my named report including any attachments I upload will be posted on the website along with the authors' responses. I agree for my report to be made available under an Open Access Creative Commons CC-BY license (http://creativecommons.org/licenses/by/4.0/). I understand that any comments which I do not wish to be included in my named report can be included as confidential comments to the editors, which will not be published.

Choose an item.

To further support our reviewers, we have joined with Publons, where you can gain additional credit to further highlight your hard work (see: https://publons.com/journal/530/gigascience). On publication of this paper, your review will be automatically added to Publons, you can then choose whether or not to claim your Publons credit. I understand this statement.

Yes Choose an item.
