# Supplementary material for: Workflow sharing with automated metadata validation and test execution to improve the reusability of published workflows
Source: Gigascience. 2023 Feb 22;12:giad006. doi: 10.1093/gigascience/giad006 (PMC9944229; doi:10.1093/gigascience/giad006)
Supplement: giad006_Reviewer_3_Report_Revision_1 — Alban Gaignard -- 11/22/2022 Reviewed [file giad006_reviewer_3_report_revision_1.pdf]

Reviewer Report

Title: Workflow sharing with automated metadata validation and test execution to improve the reusability of published workflows

Version: Revision 1 **Date:** 11/22/2022

Reviewer name: Alban Gaignard

Reviewer Comments to Author:

The reading of the revised paper would have been easier by providing updates in a different color but thank you for taking into account the comments and remarks, and clearly answering the raised issues. I also appreciated the extension of the discussion.
However, I still have some concerns regarding the proposed approach.
The proposed platform targets both workflow sharing and testing. It is explicitly stated in the abstract: "the validation and test are based on the requirements we defined for a workflow being reusable with confidence". It is clear in the paper that tests are realized through the GitHub CI infrastructure, possibly delegated to a WES workflow execution engine. Although I inspected Figure 3 as well as the wf_params.json and wf_params.yml provided in the demo website. It doesn't seem to be enough to answer questions such as: how are specified tests ? How can a user inspect what has been done during the testing process ? What is evaluated by the system to assess that a test is successful ?
I tried to understand what was done during the testing process but the test logs are not available anymore ([Add workflow: human-reseq: fastqSE2bam Â· ddbj/workflow-registry@19b7516 Â· GitHub](https://github.com/ddbj/workflow-registry/actions/runs/2257134260))
Regarding the findability of the workflows, in line with FAIR principles, the discussion mentions a possible solution which would consists in hosting and curating metadata in another database. To tackle workflow discoverability between multiple systems, accessible on the web, we could expect that the Yevis registry exposes semantic annotations, leveraging Schema.org (or any other controlled vocabulary) for instance. This would also make sense since EDAM ontology classes are referred to in the Yevis metadata file (https://ddbj.github.io/workflow-registry-browser/#/workflows/65bc3bd4-81d1-4f2a-8886-1fbe19011d81/versions/1.0.0).

Methods

Are the methods appropriate to the aims of the study, are they well described, and are necessary controls included? Choose an item.

Conclusions

Are the conclusions adequately supported by the data shown? Choose an item.

Reporting Standards

Does the manuscript adhere to the journal's guidelines on minimum standards of reporting? Choose an item.

Choose an item.

Statistics

Are you able to assess all statistics in the manuscript, including the appropriateness of statistical tests used? Choose an item.

Quality of Written English

Please indicate the quality of language in the manuscript: Choose an item.

Declaration of Competing Interests

Please complete a declaration of competing interests, considering the following questions:

- Have you in the past five years received reimbursements, fees, funding, or salary from an organisation that may in any way gain or lose financially from the publication of this manuscript, either now or in the future?
- Do you hold any stocks or shares in an organisation that may in any way gain or lose financially from the publication of this manuscript, either now or in the future?
- Do you hold or are you currently applying for any patents relating to the content of the manuscript?
- Have you received reimbursements, fees, funding, or salary from an organization that holds or has applied for patents relating to the content of the manuscript?
- Do you have any other financial competing interests?
- Do you have any non-financial competing interests in relation to this paper?

If you can answer no to all of the above, write 'I declare that I have no competing interests' below. If your reply is yes to any, please give details below.

I declare that I have no competing interests

I agree to the open peer review policy of the journal. I understand that my name will be included on my report to the authors and, if the manuscript is accepted for publication, my named report including any attachments I upload will be posted on the website along with the authors' responses. I agree for my report to be made available under an Open Access Creative Commons CC-BY license (http://creativecommons.org/licenses/by/4.0/). I understand that any comments which I do not wish to be included in my named report can be included as confidential comments to the editors, which will not be published.

Choose an item.

To further support our reviewers, we have joined with Publons, where you can gain additional credit to further highlight your hard work (see: https://publons.com/journal/530/gigascience). On publication of this paper, your review will be automatically added to Publons, you can then choose whether or not to claim your Publons credit. I understand this statement.

Yes Choose an item.
